# Evaluating the Pharmacoeconomic Impact of Nutrient Supplementation Post-operatively on Patients Receiving Roux-Y Gastric Bypass vs. Biliopancreatic Diversion with Duodenal Switch

**DOI:** 10.1007/s11695-021-05268-2

**Published:** 2021-03-17

**Authors:** Fiona A. van Vollenstee, Maria T. van der Merwe

**Affiliations:** 1 Centre of Excellence of Metabolic Medicine and Surgery (CEMMS), Johannesburg, South Africa; 2 School of Medicine, Department of Health Sciences, University of Pretoria, Pretoria, South Africa

**Keywords:** Bariatric surgery, Pharmacoeconomics, Costs, Supplementation, Nutrient deficiencies, TPN, costings

## Abstract

**Background:**

Without the needed medical support, bariatric surgery can be associated with post-operative malnutrition and associated nutrient deficiencies. We aimed to evaluate the cost difference of perioperative infusion requirements and TPN between GBP and BPD-DS.

**Methods:**

All patients undergoing GBP or BPD-DS procedures between August 2015 and June 2018 were included. Information was collected to standardize the nutritional information into two categories: (1) oral supplementation and standard intravenous infusions, as predicted costs forming part of preoperative quote and (2) infusions prescribed for malnutrition, based on blood biochemistry, caterized as unexpected costs.

**Results:**

A total of 573 patients over 3 years (GBP 60%, BPD-DS 40%) were included in the analysis. The average predicted costs from oral supplementation for both surgery groups and prophylactic infusions for BPD-DS were GBP (46.90USD) vs. BPD-DS (154.13 USD) (*p-*value = NS). Unexpected costs for infusions to correct nutritional deficiencies were GBP (199.14 USD) vs. BPD-DS (127.29 USD) (*p-*value = NS). TPN incidence rate was GBP (2.1%) and BPD-DS (12.7%) (*p*-value < 0.001) and admission rate per patient was GBP (0.9) and BPD-DS (0.63) (*p*-value < 0.05). Costs for acquiring TPN were GBP (153.58 USD) vs. BPD-DS (268.76 USD). Total unexpected costs were GBP (352.72 USD) vs. BPD-DS (396.05 USD) (*p-*value = NS).

**Conclusion:**

Nutrient deficiencies are known to occur within both GBP and BPD-DS surgeries, even up to 3 years. The admission rate/patient, requiring TPN, was higher in the GBP group, indicating that BPD-DS surgery can be efficient and cost-effective with holistic and multitherapeutic post-surgery care. BPD-DS procedures should be reserved for centers with a comprehensive and experienced multidisciplinary team enforcing stringent follow-up regimes.

## Introduction

Obesity remains a global and growing concern in both developed and developing countries. The obesity burden is related to the chronic, relapsing, and progressive nature of the disease together with the various associated co-morbidities. The Non-communicable Disease Risk Factor Collaboration predicted obesity prevalence rates to reach 18% in men and 21% in women globally by 2025. Higher prevalence rates are predicted among South Africans with men reaching up to 25.6% and women up to 47.7% [1].

Bariatric surgery (metabolic surgery) has been shown to be revolutionary towards resolving obesity-related co-morbidities either directly or indirectly. Frequently observed co-morbidities of obesity include type II diabetes mellitus (T2DM), sleep apnoea, hypertension, hyperlipidaemia, depression, ischaemic heart disease, NASH, and various cancers. These co-morbidities develop and progress with the duration and progression of the obesity disease status.

The effect of bariatric surgery on food intake includes various changes in hormonal, altered physiological mechanisms, and calorie wasting in some procedures. Sustainable effects on weight reduction will result from changes in gastrointestinal hormone secretion, energy expenditure, bile acid metabolism, intestinal bacterial colonization, and epigenetic changes [2, 3].

**Fig. 1:**
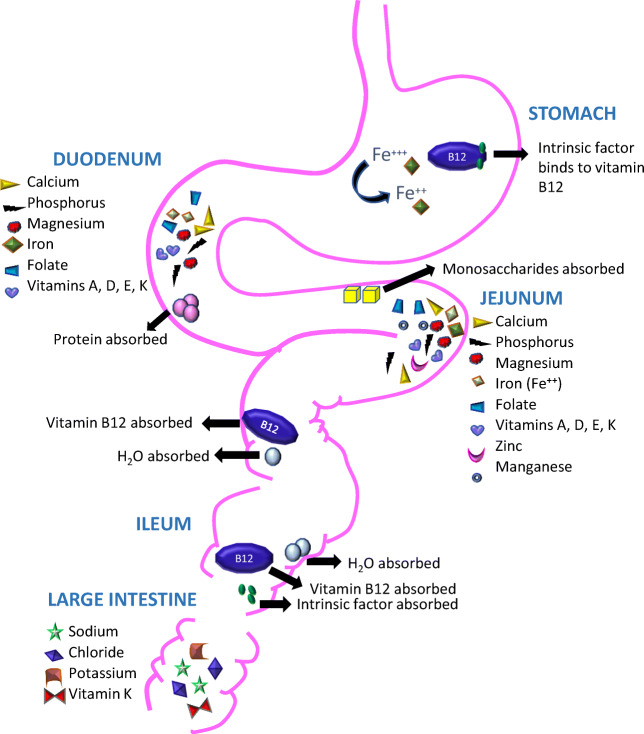
Representation of the gastrointestinal tract indicating the site where specific elements are absorbed or converted for absorption. Bariatric surgery procedures are associated with various nutrient deficiencies that are related to the affected anatomical site influencing normal digestion and absorption

Bariatric surgery procedures are associated with a post-operative malnutrition state and decrease in the absorption of specific nutrient elements. This is due to some of these elements being converted and absorbed in different locations within the gastrointestinal track (Fig. 1). These nutrient elements include fat-soluble vitamins (vitamins A, D, E, and K), trace elements, vitamin B12, folic acid, calcium, albumin, and iron [4–7].

Optimizing post-surgery nutritional status begins pre-operatively with normalizing nutrient deficiencies, increasing protein intake, and extensive dietary counselling with the patient. Both GBP and BPD-DS procedures will require stringent macro- and micronutrient monitoring to detect deficiencies and supplement patients accordingly. Guidelines of nutrient deficiency monitoring and supplementation have been described and amended with the growing field of metabolic surgery [4–7].

Comparisons of medical costs of obese patients receiving bariatric surgery vs. conventional treatment have been well described, but little is known about the post-operative cost-to-care between the different bariatric surgery procedures. A worldwide perception exists that BPD-DS procedures are costlier and more labor intensive than GBP. At our center, nutrient deficiencies are treated with both oral supplementation as well as infusions in anticipation and with a view to replenishing nutrient deficiencies determined by blood pathology results. Different nutrient replacement strategies apply for both GBP and BPD-DS surgeries, based on an understanding of the anatomical changes of each surgical procedure. Differences in incurring cost can therefore also be anticipated and more carefully calculated in the different procedure types offered. To date, supplementation requirements and associated costs have not been well documented and may be an unexpected expense in the patient follow-up that was not included in the initial expense budget. In addition, comparative data on supplementation needs between the different procedures are also very limited and to the best of our knowledge have never been calculated and reported on before.

Financial and economic impact comparisons between bariatric surgery and conventional treatment of patients with obesity have been extensively evaluated. Although many studies mention the costs impact of different surgeries, to our knowledge, little have been reported on the direct comparison of costs involved between GBP and BPD-DS post-surgical care. The aim of the study was to evaluate the pharmacoeconomic impact of post-surgical nutritional status and supplementation requirements of bariatric surgery patients receiving either GBP or BPD-DS. In addition, we evaluated the cost-effectiveness of our standard regimes of outpatient intravenous replacement for BPD-DS patients in the first 12 months post-surgery with view to limiting hospital admissions and preventing chronic malnutrition.

## Material and Methods

The study was performed at Netcare Waterfall Hospital in South Africa. Ethical approval was obtained from the Faculty of Health Sciences Research Ethics Committee of the University of Pretoria (205/2018). The Rand to Dollar exchange rate ($1 USD = 15.18 ZAR) was calculated on 03/09/2019 at 12 pm according to XE Currency Converter (www.xe.com).

A retrospective analysis was performed on all patients undergoing GBP or BPD-DS procedures between 1 August 2015 and 30 June 2018. All non-compliant patients not adhering to stringent follow-up regimens have been excluded from the study.

Various criteria were taken into consideration for surgery type indication. A BMI > 47 kg/m^2^ was used for allocation of a BPD-DS surgery and a BMI > 35 kg/m^2^ with two or more co-morbidities would qualify for either GBP or BPD-DS surgery [4–7]. To exclude possible variations, all surgeries were overseen and performed by a single surgeon and endocrinologist with more than 20 years’ experience. The GBP involves a gastric pouch reconstruction (15–30 ml), with a 30-cm afferent limb and alimentary channel ranging between 120 and 180 cm. The BPD-DS procedure involves a sleeve gastrectomy (100 ml), with an alimentary limb length of 200 cm and mean common channel length of 110 cm with range between 80 and 150 cm [8–10].

Clinical and nutritional status was determined using extensive blood chemistry results. For the purpose of adding value to the manuscript, only the following variables were reported: weight (kg), body mass index (BMI) (kg/m^2^), fasting triglycerides (mmol/L), vitamin D, (ng/ml), vitamin A (μg/L), vitamin E (mg/L), ferritin (ng/L), vitamin B12 (g/L) calcium (mmol/L), red cell folate (nmol/L) as well as albumin (g/L) (Table 1). Deficiencies were compared between the two surgical cohorts at both pre-surgical and post-surgical (1 and 2 years) intervals. Statistical differences were assessed for each parameter between GBP and BPD-DS using the unpaired *t*-test method and a *p-*value < 0.05 was regarded as significant.

**Table 1:** Outcome data the nutritional status of GBP vs. BPD patient cohort (Aug 2015–Jun 2018)

Parameter reference range	GBP baseline (*n* = 344)	BPD baseline (*n* =229)		GBP 12MO (*n* = 206)	BPD 12MO (*n* = 112)	GBP 2YR (*n* = 93)	BPD 2YR (*n* = 33)	
Weight kg	116.16 ± 0.93	153.15 ± 2.05	**###**	81.98 ± 0.98	94.38 ± 1.79 **###**	79.73 ± 1.40	87.26 ± 2.60	**##**
BMI kg m^−2^	41.62 ± 0.22	52.97 ± 0.57	**###**	29.31 ± 0.27	32.26 ± 0.54 **###**	28.63 ± 0.40	29.47 ± 0.72	*0.28*
Vitamin D ng/ml RR: > 20	21.44 ± 0.45	22.10 ± 0.99	*0.50*	60.20 ± 2.07	40.68 ± 1.80 **###**	63.25 ± 7.08	34.16 ± 2.74	**#**
Vitamin A μg/l 300–800	596.09 ± 10.13	564.39 ± 10.84	**#**	583.35 ± 12.07	466.49 ± 15.62 **###**	617.11 ± 19.90	438.02 ± 35.79	**###**
Vitamin E mg/l 5.5–18	13.47 ± 0.24	12.66 ± 0.26	**#**	11.91 ± 0.22	9.31 ± 0.23 **###**	12.15 ± 0.30	8.57 ± 0.38	**###**
Ferritin ng/l RR: 13–150	136.72 ± 8.18	197.48 ± 15.95	**##**	138.47 ± 8.74	285.59 ± 20.63 **###**	145.43 ± 12.43	254.39 ± 31.40	**##**
Vitamin B12 g/l RR: 107–443	323.93 ± 8.13	330.71 ± 11.61	*0.62*	314.76 ± 10.47	432.11 ± 18.51 **###**	352.61 ± 17.38	486.83 ± 34.32	**##**
Calcium mmol/l RR: 2.15–2.50	2.31 ± 0.01	2.31 ± 0.01	*0.93*	2.31 ± 0.01	2.29 ± 0.01 **#**	2.69 ± 0.40	2.28 ± 0.02	*0.56*
Albumin g/l RR: 35–52	39.92 ± 0.19	39.35 ± 0.23	*0.06*	40.31 ± 0.22	38.16 ± 0.39 **###**	40.64 ± 0.47	40.09 ± 0.67	*0.53*
Red Cell Folate nmol/l RR: 317–1894	1579.98 ± 35.54	1568.88 ± 40.53	*0.84*	2153.66 ± 45.13	1739.16 ± 57.38 **###**	2069.98 ± 56.31	1814.89 ± 83.75	**##**

Values expressed in mean ± SEM

*P*# < 0.05; *P*## < 0.01; *P*### < 0.0001; unpaired *t*-test GBP vs.

The numbers in italics is the statistical significance (*p* < 0.05)

### Malnutrition

All patients received clinician prescribed oral supplementation as part of standard care according to post-surgery supplement recommendations [6]. Patient received 1 of 10 different standardized combinations containing B-Cal Ultra tablets, Calciferol 50,000 IU, ActivoVite™ Pro4, Astyfer® capsules, Activo Vitamin B12 Spray, and Calcium Citrate D® (Table 2). The composition of respective oral supplements is summarized below (Table 2). The average cost of oral supplementation per patient per procedure was calculated and assessed for statistical difference. Due to the dangers of vitamin A deficiency and over supplementation of vitamin A becoming toxic to the body, additional retinoic vitamin A oral supplementation was given to BPD-DS, with severe blood biochemistry deficiency.

**Table 2:** Dosage range of the different oral supplement packages and the composition of the respective supplementation. The infusion supplementation types and composition per respective infusion

Oral supplementation	Dosage range	Composition of one tablet/spray
B-Cal Ultra Tabs	1xday: *mane*	Calcium carbonate (500 mg), vitamin D (400 IU), magnesium (85 mg), copper (1 mg), folic acid (490 μg), and manganese (2 mg)
Activovite™ Pro4	1xday: *mane*	Multivitamins, minerals and probiotic: retinyl acetate (900 μg), thiamine (3.6 mg), riboflavin (3.9 mg), nicotinamide (25 mg), pantothenic acid (5 mg), pyridoxine (5.1 mg), biotin (90 μg), folic acid (400 μg) and cyanocobalamin (7.2 μg), vitamin C 50 mg, vitamin D (15 μg), vitamin E (15 mg), calcium phosphate (60 mg), chromium (35 μg), copper (0.9 mg), iron (9 mg), magnesium (84 mg), manganese (1.15 mg), molybdenum (45 μg), phosphorus (47 mg), selenium (55 μg), zinc (55 μg) and probiotics (2.5 × 10^8^) of *Lactobacillus rhamnosus*, *Lactobacillus salivarius*, *Bifidobacterium lactis* and *Bifidobacterium longum*
Activo Vitamin B12 Spray	1xday: bedtime	1 spray contains: methylcobalamin (300 μg) and chromium (10 μg)
Calcium Citrate D® Eff	1xday: *mane*	Elemental calcium (500 mg), Vitamin D3 (400 IU)
Calciferol 50,000 IU	1×week 2×week 3×week 5×week OR 1×day: *mane*	Vitamin D (50,000 IU)
Folic acid OR	1xday: *nocte*	Folic acid 5 mg/day
Astyfer®	1xday: *nocte*	Ferrous fumarate (150 mg)
Infusion type	Per single infusion	Infusion composition
Vitalipid®	1× infusion	Vitamin A 3300 IU, vitamin D_2_ 20 IU, vitamin E 1 IU and K_1_ 15 μg
Vitalipid® with Cernovit ^TM^ (V+C)	1× infusion	Vitamin A 3500 IU, vitamin D_3_ 200 IU, vitamin E 11.2 IU, vitamin C 125 mg, vitamin B_3_ 46 mg, vitamin B_5_ 17.25 mg, vitamin B_6_ 4.53 mg, vitamin B_2_ 4.14 mg, vitamin B_1_ 3.51 mg, folic acid 414 mcg, D-biotin 60 mcg, vitamin B_12_ 5.5 mcg and DL α-tocopherol 10.2 mg
Additrace®	1× infusion	Ferric chloride 20 μmol, zinc chloride 100 μmol, manganese chloride 5 μmol, copper chloride 20 μmol, chromic chloride 0.2 μmol, sodium selenite anhydrous 0.4 μmol, sodium molybdate 0.2 μmol, sodium fluoride 50 μmol and potassium iodide 1 μmol
Soluvite®	1× infusion	Vitamin B_1_ 3.2 mg, vitamin B_2_ 3.6 mg, nicotinamide 40 mg, vitamin B_6_ 4 mg, pantothenic acid 15 mg, vitamin C 100 mg, biotin 60 μg, folic acid 0.4 mg and vitamin B_12_ 5 μg
Venofer®	1× infusion	300 mg iron and 4.35 g sucrose

The total amount of patient infusions per procedure were calculated and stratified according to type. The average amount of infusions required per patient per procedure was established and subsequently the average cost per infusion per patient per type of procedure was established. Associated costings were further categorized into two groups: (1) predicted cost (fixed cost) and (2) unexpected cost. Predicted cost included the oral supplementation cost for both surgical groups as well as any planned infusions for BPD-DS patients (CEMMS protocol). The standard treatment for BPD-DS patients was to receive prophylactic infusions Vitalipid® or Vitalipid® with Cernevit^TM^ (V+C) infusions perioperative, 2 months and 6 months, based on blood biochemistry. From 12 months onwards, infusions were prescribed when blood chemistry deficiency was identified.

Criteria for nutrient supplementation and replacements have been well described in consensus documents [4–7]. Indication for additional infusions was prescribed by a single endocrinologist based on clinical judgement and with consideration of nutrient deficiencies revealed by routine blood biochemistry. The indication for this was when oral supplementation was of poor tolerance by patient, insufficient diet intervention, or replacement of nutrients needed due to surgical complications. These infusions were considered as unpredicted/unexpected costs, and include Additrace®, Soluvite®, and Venofer® (Fig. 3). The composition of the respective infusions is presented in Table 2.

All infusion treatments were performed in the outpatient-based clinic setting, where patients were treated under the supervision of a registered nurse and at substantially lower rates compared to in-hospital treatment. A comparison was drawn between costs incurred per infusion in the outpatient infusion clinic and a standard hospital admission.

### Protein Deficiency

The risk for requiring total parenteral nutrition (TPN) among the 2 cohorts was determined from January 2014 until December 2016. Indication of TPN was serum albumin levels < 25 g/L and life-threatening conditions due to unexpected chronic nausea syndrome or bowl obstruction. At least one or more Nutriflex infusions (N4-600 with electrolytes or N9-840 with electrolytes), Baxter Healthcare South Africa (Pty) Ltd, were administered per TPN admission. The TPN admission rate was calculated per cohort by dividing the number of TPN admissions by the number of patients and compared for statistical difference. The median quantity of TPN administrations required on the first admission was determined. The total cost for TPN was calculated by multiplying the TPN admissions, the median TPN required quantity, and the known cost per TPN administration (Fig. 4).

The average predicted, unexpected, and total estimated cost per procedure was calculated and a cost comparison between the two surgical cohorts was performed. All statistical analysis was performed using the unpaired *t*-test comparing GBP to BPD-DS. Statistical significance was deemed a *p*-value < 0.05.

## Results

A total of 573 patients received bariatric surgery during the study period and were included for analysis. The patients were divided into two cohorts based on the type of surgery received with GBP patients being 60% (*n*=344) of the total patients, and BPD-DS patients being 40% (*n* = 229) of the total cohort.

### Malnutrition

Significant differences in the nutrient status of the patients in the two surgical cohorts were observed post-surgery with the GBP patients displaying significantly lower levels of ferritin and vitamin B12 compared to BPD-DS patients. In comparison, BPD-DS patients had lower levels of fat-soluble vitamins and serum albumin at 12 months. No significance (*p*-value = NS) for these indices was present between GBP vs. BPD-DS patients by 24 months (Table 1).

Oral supplementation was regarded as a fixed post-operative cost for both cohorts. The 10 standardized packages varied with regard to cost and prescribed frequency of respective supplementation (Table 2). The average predicted cost of oral supplementation was marginally different for GBP (46.90 USD) and BPD-DS (48.14 USD) (*p*-value = NS) cohorts. No significant cost difference with regard to oral supplementation between the two cohorts was observed (*p*-value = 0.976) (Table 2).

All the BPD-DS patients received prophylactic infusions as part of routine care consisting of fat-soluble vitamins and trace elements at time of surgery, 2 months and 6 months to prevent malnutrition in the early post-operative time when inadequate intake of food volume and food spectrum exists. These infusions were considered to form part of the predicted costs for patients receiving BPD-DS surgery and were estimated at 105.99 USD excluding the outpatient facility fee (Table 3).

**Table 3:** Medication cost per patient receiving infusions with the unpredicted/unexpected cost as well as the predicted/fixed costs for BPD-DS patients

Medication	Average infusions per patient		Medication cost	Average cost per infusion per patient (USD)	
	GBP	BPD-DS	USD	GPB	BPD-DS
ADDITRACE® [GBP (*n* = 12); BPD-DS (*n* = 8)]	2.0	1.5	10.95	21.91	16.43
CERNEVIT [GBP (*n* = 25); BPD-DS (*n* = 18)]	1.6	1.5	14.41	22.47	21.61
SOLUVIT [GBP (*n* = 8); BPD-DS (*n* = 8)]	1.8	1.1	13.00	22.75	14.69
VENOFER® [GBP (*n* = 128); BPD-DS (*n* = 111)]	2.0	1.6	36.11	72.22	57.41
VITALIPID®[GBP (*n* = 15); BPD-DS (*n* = 4)]	1.5	1.0	11.99	18.35	12.25
V+C [GBP (*n* = 69); BPD-DS (*n* = 10)]	1.6	0.2	26.40	41.48	4.91
Unpredicted/unexpected (Excluding TPN)				199.14 USD	127.29 USD
Costs expected for TPN/patient				153.58 USD	268.76 USD
Total unpredicted/unexpected (Including TPN)				352.72 USD	396.05 USD
VITALIPID®[BPD-DS prophylactic infusions]	3.49	NA	11.99	NA	41.85
V+C [BPD-DS prophylactic infusions]	2.43	NA	26.40	NA	64.14
Predicted/fixed cost					105.99 USD

To consider cost efficiency to third party funders, an outpatient facility was used for administration of infusion by and under supervision of a registered nurse. Using an outpatient-based infusion clinic is 81.95 USD vs. in-hospital infusion is 284.39 USD is effective and cost efficient.

**Fig. 2:**
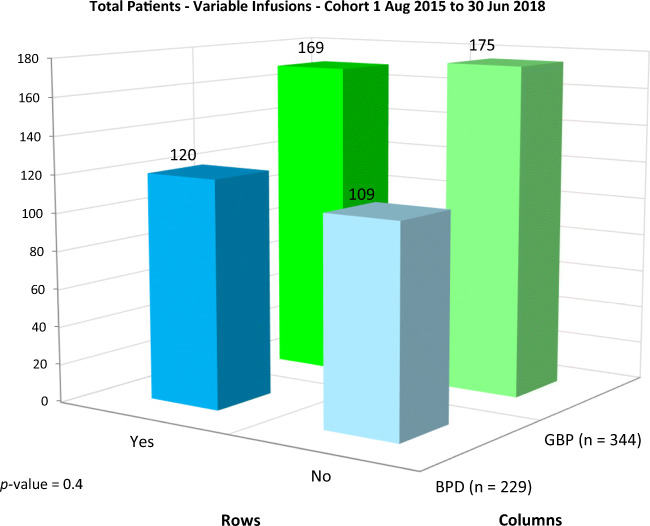
Total amount of patient receiving metabolic surgery between August 2015 and June 2018

**Fig. 3:**
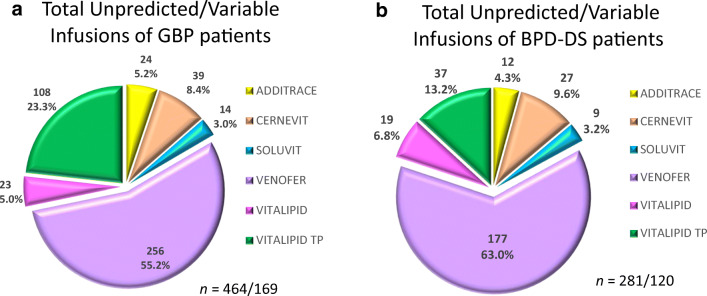
Pie charts of the total unpredicted/unexpected infusions for GBP patients (**a**) and BPD-DS patients (**b**). These infusions were prescribed based on blood pathology investigations

Other infusions (unexpected cost) were required in 169 (49.1%) of GBP and 120 (52.4%) of BPD-DS patients over 3 years, displaying no significant difference (*p*-value = NS) (Fig. 2). The total amount of infusions required in the GBP group (*n* = 464) vs. the BPD-DS group (*n* = 281) were significantly higher (*p*-value < 0.01) for iron, trace minerals, and fat-soluble vitamins (Table 4). Venofer® was the most frequently prescribed infusion in both cohorts, for correction of iron deficiency (Figs. 2 and 3). GPB procedure patients were more prone to develop food volume associated deficiencies early in the post-operative period up to 3 years, requiring a higher amount of Vitalipid® and Cernevit (V+C) (*p-*value < 0.001) and Venofer® (*p-*value 1 < 0.00001) compared to BPD-DS patients (Table 4).

**Table 4:** Initial (first) infusions administered to BPD vs. GBP patients

	Unexpected infusions			% Pt unexpected infusions	
Medication	GBP	BPD-DS	*p*-value	GBP	BPD-DS
	*(n* = 169/344)	(*n* = 120/229)		(*n* = 344)	(*n* = 229)
ADDITRACE®	24	12	0.585	7%	5.2%
CERNEVIT	39	27	0.575	7.3%	11.8%
SOLUVIT	14	9	0.887	4.1%	3.9%
VENOFER®	256	177	< 0.00001	74.4%	77.3%
VITALIPID®	23	19	0.3	6.7%	8.3%
V+C	108	37	< 0.001	31.4%	16.2%
Total unexpected infusions	464	281

The average amount of infusions per patient were higher for GBP (199.14 USD) than BPD-DS (127.29 USD) patients (*p*-value = 0.32; *p-*value = NS). The estimated cost saving per patient receiving infusion treatment in the outpatient clinic-based setting compared to in-hospital amounted to ± 200 USD per day.

### Protein Deficiency

**Fig. 4:**
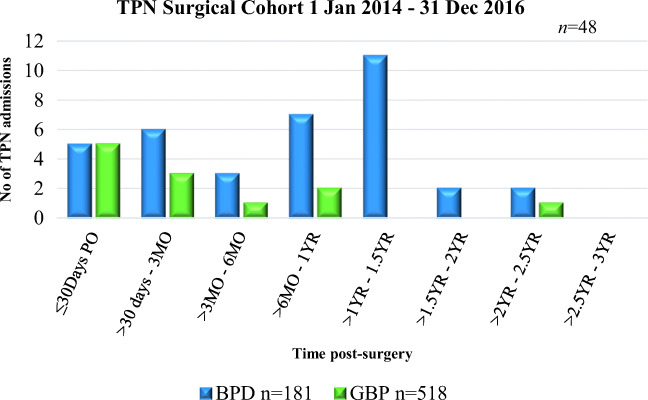
No of TNP admissions (*n* = 48) required at certain time intervals of patients receiving respective bariatric surgery from January 2014 up to December 2016. In the GBP cohort (*n* = 518) total of 12 admissions were required among 11 patients and in the BPD-DS cohort (*n* = 181) 36 admissions among 23 patients

The need for requiring TPN was calculated at an incidence rate of 2.1% in the GBP group vs. 12.7% in the BPD-DS group (*p*-value < 0.001); however, the TPN admission rate per patient was higher in GBP (0.9) vs. BPD-DS (0.63) (*p-*value < 0.05). The median quantity of required TPN per first admission was lower in GBP (2%) vs*.* BPD-DS (5%) (Fig. 4). The known cost per TPN administration was 85.32 USD. The mean predicted cost for TPN was significantly lower for GBP (153.58 USD) vs. BPD-DS (268.76 USD) (*p-*value < 0.0001). TPN admissions are an unforeseen cost that is often associated with late surgical complications and an unpredicted/unexpected cost that could be incurred by these patients.

The average predicted cost was 46.90 USD in GBP vs. 154.13 USD in BPD-DS. The mean when combining the predicted and unpredicted/unexpected costs showed no significant difference with 352.72 USD for GBP and 396.05 USD (*p*-value = NS) for BPD-DS patients.

### Discussion

The impact of bariatric surgery on healthcare costs has been described for both incurring costs post-operatively regarding nutritional supplementation and overall cost saving over time by decreasing various co-morbidities [11]. Obese patients (BMI > 30) have a 30% higher healthcare cost compared to normal and non-obese patients (BMI 18.5–24.9) [12]. Obese patients in the USA incur on average 1114.69 USD more to medical spending than normal patients [13]. The return on investment for laparoscopic-based bariatric surgery procedures is recovered after 2–3 years of surgery [14, 15]. This cost saving is related to reduction in prescription drug use, physician visits, and hospitalization costs (including emergencies). The long-term cost saving following bariatric surgery is associated with the reduction in co-morbidities including T2DM, coronary heart disease, depression, hypertension, and OSA [15]. Greater economic impact can be observed with bariatric patients with co-morbidities especially T2DM [16, 17]. Surgical and post-operative cost saving was demonstrated by comparing obese patients receiving bariatric surgery to those receiving conventional treatment [12–14]. After 5 years, an absolute difference in medical expenditure was 8,054,285.31 USD per 1000 patient cohort [14]. Considering the rising obesity epidemic, exponential increase in estimated costs could be expected with every 1-unit BMI increase and will result in up to a 4% increase in overall medical cost and 7% increase in pharmaceutical costs [18].

Indirect costs associated with obesity include absenteeism and presenteeism (reduced productivity at work) [13, 14]. Moreover, obese patients presenting with co-morbidities will result in higher *pro rata* medical costs, with a 1-unit BMI increasing cost by 20%, but in contrast, a 1-unit BMI loss decreasing overall cost by only 8% [19]. By implication, it is therefore important to factor in all predictable and unpredictable post-operative medical costs that patients could incurr based on clinical observation. Considering the direct medical costs of associated co-morbidities and complications together with indirect costs caused by work absence, reduced productivity, disability, and premature death, bariatric surgery has been favored as a cost-effective and cost-saving treatment option for obese patients due to a higher and more sustainable weight loss [20]. We are unaware of any post-operative cost calculations of maintaining health in GBP and BPD-DS patients in a post-operative setting.

A position statement released in 2016 by the American Society for Metabolic and Bariatric Surgery on long-term survival benefits following surgery indicated that bariatric/metabolic surgery (1) reduces all-cause mortality and increases long-term survival; (2) is superior to best medical management and treatment of type 2 diabetes mellitus (T2DM) and reduces T2DM associated deaths; (3) reduces cardiovascular disease (CVD) risk factors as well as future events and deaths; and (4) reduces risks of certain cancers and improves cancer outcomes and associated deaths especially in females [21]. It is self-explanatory that these health economic benefits can diminish rapidly in the presence of poor care and poor worldwide follow-up of bariatric patients.

The aim of the present study was to highlight the difference in general costs involved to maintain health after GBP and BPD-DS surgery. Financial and economic impact comparisons between bariatric surgery and conventional treatment of patients with obesity have been extensively evaluated. Although many studies mention the costs impact of different surgeries, to our knowledge little have been reported on the direct comparison of costs involved between GBP and BPD-DS post-surgical care.

Nutrient deficiencies were tested prior to surgery and normalized with respective infusions, as suggested by Strain et al., allowing normalization of nutrient status at baseline [22]. Due to the anatomical changes required and malabsorptive nature in both GBP and BPD-DS surgeries, nutrient deficiencies will be eminent. Lower levels of iron and vitamin B12 were found in GBP patients compared to lower levels of fat-soluble vitamins in the BPD-DS patients. The pouch formation of the GBP is causative of the lack of iron and vitamin B12, as Fe+3 does not convert to absorbable Fe+2 or intrinsic factor not binding to vitamin B12 in the stomach for down-stream absorption [23]. Fat-soluble vitamins need lipase and bile salts to aid with absorption process. In the BPD-DS, anatomical changes will bypass the two major sites for fat-soluble vitamin absorption in the duodenum and jejunum, causing more frequent fat-soluble vitamin deficiencies (Fig. 1) [2, 24].

Both surgeries demonstrate vitamin B12 deficiency due to either the pouch formation or sleeve resection in the GBP and BPD-DS, respectively. Although oral supplementation was prescribed for all bariatric patients, compliance is difficult to regulate [22]. Infusion intervention provided a more structured environment of replacement by professional staff, had the additional advantage of underwriting the seriousness of replacement, and also afforded the professional team an additional opportunity and platform for patient education. Infusions were used where oral supplementation failed at maximum dose and deficiency became life threatening. All patients were on oral supplementation and prophylactic intravenous infusions for BPD-DS cohort, and where no progress were observed with increasing the respective supplementation, the endocrinologist reverted to infusions for correcting nutrient deficiencies.

The average cost of oral vitamin supplementation was not significantly different between the two groups. The predicted infusion cost of 105.99 USD for the BPD-DS patient is set out at baseline to patients and medical funders in order to keep the ensuing unexpected cost in this cohort down to a negligible amount. The BPD-DS patients become less labor intensive after 18 months and more cost-effective with an average total infusion cost per patient of 127.29 USD compared to 199.14 USD for the GBP patients. A major cost driver for both cohorts was related to iron supplementation with Venofer® infusions, highlighting the high degree of iron intolerance and malabsorption associated with both procedures. Off note, fat-soluble vitamin deficiencies were more frequent among the GBP patients contributing a significant cost factor, in contrast to what has been published in the medical literature to date. The impact of portion size, chronic nausea syndrome, and intolerability of certain food may be a factor to consider in GBP procedures.

Treatment in the outpatient clinic setting is highly cost-effective with a saving of 202.44 USD for nutrient replacement infusions per patient visit. The benefits include a significant cost saving to medical aids, increasing service availability. In addition, it provides easy, comfortable, and quick access for the patient.

Extensive dietary counselling before and after surgery forms part of basic level of care. Protein intake is guided by a registered dietician trained in the field of bariatrics, where every patient is assessed on an individual basis according to age, gender, weight, and dietary goals and nutritional requirements were calculated according to international guidelines [5, 25]. A high protein soft diet aiming at > 80 g protein a day is followed 1 week prior to surgery. Dietary recommendations for post-surgery GBP patients aim for a minimum of 60 g protein per day to 1.2–1.5 g/kg ideal body weight per day. Where BPD-DS male and female patients were advised to a minimum of 100 g and 80 g protein per day respectively up to 1.5 g/kg ideal body weight. To reduce loss of lean body mass in patients rapid losing weight, protein requirements could be up to 2.1 g/kg ideal body weight per day.

Long-term complications of bariatric surgery may include protein malnutrition or hypoalbuminemia. Intravascular serum albumin level of < 35 g/L (hypoalbuminemia) has been used as an indicator of protein malnutrition, where level < 25 g/L was considered life threatening and an indication for in-hospital TPN administration. Although many factors could be the cause of low albumin levels including liver disease, sepsis, and nephrotic syndrome [26], all complications in this study were caused by chronic nausea syndrome or obstructive bowl syndrome.

In this study, the risk for requiring TPN up to 18 months post-operative was significantly higher in the BPD-DS group; however, the admission rate was significantly lower. The amount of TPN infusions required per patient was higher and added to the unpredicted cost difference of 115.18 USD. A decrease in TPN admission over time after surgery was observed in the GBP cohort. Possible reasons for an increase in TPN admissions after 18 months could be due to the non-compliancy to diet, poor adherence to supplementation, as well as a relaxation in patient attitude to life-style changes that is required with bariatric surgery. The risk acquiring TPN was higher in the BPD-DS cohort up to 18 months post-surgery. This could be due to higher malabsorptive qualities of the procedure, which is in correlation with low serum albumin levels observed at 12 months, or nausea and diarrhea from picking food with hidden fats during the early learning curve.

Although GBP is considered the “gold-standard” of bariatric surgery, BPD-DS is still one of the most efficacious surgical procedures in terms of excessive weight loss and reduction in co-morbidities [27–29]. Due to the complex Scopinaro technique of the BPD-DS surgery, it is not widely used. With the acceptance of the mini gastric bypass-one anastomosis gastric bypass (MGB-OAGB) as a safe and effective surgery, a slow and constant drift towards this more recent procedure can be expected [30, 31]. The MGB-OAGB is also associated with surgical complications like sleeve staple line, duodenal stump, and duodenoileostomy leaks. These leaks could be more problematic than other procedures because of the presence of bile [32]. Although the sleeve gastrectomy and GBP are currently the more common weight loss surgery, procedures perceived technically difficult and with greater operative risk profile, like the BPD-DS, can aid in greater weight loss and metabolic improvement, especially in patients with BMI > 50 kg/m^2^ [33]. This study also demonstrates the advantage of BPD-DS surgery for patients with high BMI > 50 kg/m^2^, regarding significant excess weight loss and reduction in BMI. Due to high surgical risk of BPD-DS surgery, it should be reserved for bariatric centers with a comprehensive and experienced multidisciplinary team enforcing stringently follow-up regimes.

One limitation to this study is the unknown long-term cost difference between the cohorts to maintain health. Loss to follow-up affects all centers globally, making long-term research challenging. More research needs to be done on cost involved rescuing the non-compliant vs. compliant patient as is the case in our patient cohort. Similarly testing all the possible nutrient parameters is affected.

## Summary and Recommendation

Current literature on care for BPD-DS patients is inadequate and does not account for good outpatient and prophylactic care of BPD-DS patients. Predicted nutrient replacement cost of BPD-DS is higher due to the prophylactic infusions given. This could be expected since BPD-DS surgery is a more severe treatment intervention with patients more prone to develop nutrient deficiencies. Oral iron intolerance is high after bariatric surgery regardless of the specific surgery and associated anatomical changes. The unpredicted costs for unexpected nutritional replacement are effectively higher in GBP (199.14 USD) vs*.* BPD-DS (127.29 USD) (*p*-value = NS). Admission rate per patient requiring TNP was higher in the GBP cohort. The BPD-DS patient can be cared for as cost efficiently as for GBP, provided that stringent follow-up protocols are in place. A significant health economic saving can be expected when running an efficient outpatient-based care and infusion clinic. Multitherapeutic post-surgery care needs to be standardized worldwide according to specific bariatric surgery, taking into consideration the health economic impact. BPD-DS procedure should be reserved for centers with a comprehensive and experienced multidisciplinary team enforcing stringent follow-up regimes.
